# Social interactions among ants are impacted by food availability and group size

**DOI:** 10.1242/bio.060422

**Published:** 2024-10-16

**Authors:** Xiaohui Guo, Matthew J. Hasenjager, Nina H. Fefferman, Noa Pinter-Wollman

**Affiliations:** ^1^ Department of Ecology and Evolutionary Biology, University of California, Los Angeles, CA 90095, USA; ^2^ Department of Ecology and Evolutionary Biology, University of Tennessee, Knoxville, TN 37996, USA; ^3^ Department of Mathematics, University of Tennessee, Knoxville, TN 37996, USA

**Keywords:** Flexibility, Food sharing, Network dynamics, Social network analysis, Supply chains, *Camponotus fragilis*

## Abstract

Social interactions are important for how societies function, conferring robustness and resilience to environmental changes. The structure of social interactions can shape the dynamics of information and goods transmission. In addition, the availability and types of resources that are transferred might impact the structure of interaction networks. For example, storable resources might reduce the required speed of distribution and altering interaction structure can facilitate such change. Here, we use *Camponotus fragilis* ants as a model system to examine how social interactions are impacted by group size, food availability, and food type. We compare global- and individual-level network measures across experiments in which groups of different sizes received limited or unlimited food that is either favorable and cannot be stored (carbohydrates), or unfavorable but with a potential of being stored (protein). We found that in larger groups, individuals interacted with more social partners and connected more individuals, and interaction networks became more compartmentalized. Furthermore, the number of individuals that ants interacted with and the distance they traveled both increased when food was limited compared to when it was unlimited. Our findings highlight how biological systems can adjust their interaction networks in ways that relate to their function. The study of such biological flexibility can inspire novel and important solutions to the design of robust and resilient supply chains.

## Teaser

**Summary:** Social interactions among ants are impacted by food availability and group size but not by food type.

## INTRODUCTION

Social interactions facilitate the flow of information, resources, and disease throughout societies. The network structures that emerge from these interactions impact how rapidly and how evenly resources are distributed throughout a population. Both global- and individual-level network features influence these dynamics. For example, networks that are highly modular and subdivided facilitate different flows compared to networks that are uniformly connected (Sah et al., 2017; Salathé and Jones, 2010; Silk and Fefferman, 2021). Similarly, individual connectivity can impact the dynamics of flow on a network, for example highly connected individuals can have a disproportionate impact on disease spread (Lloyd-Smith et al., 2005).

While much theoretical (Eames and Keeling, 2002) and empirical (Stroeymeyt et al., 2014) work has been devoted to uncovering the ways in which network structure influences transmission dynamics, especially in the context of disease transmission (Fefferman and Ng, 2007; Firth et al., 2020; Gates and Woolhouse, 2015; Godfrey, 2013; Jones et al., 2018; Pautasso and Jeger, 2008), less attention has been devoted to the way in which the nature of what is being transmitted might impact network structure. For example, foraging for resources with high spatiotemporal variability may promote food-sharing networks that are structured in a way that mitigates collective resource shortfalls (Jones and Ready, 2022). The way in which animals interact is especially important when groups have shared goals, such as social insects, in which sterile workers cooperate to produce reproductives that will found new related colonies. In such cooperative groups, the way in which resources, such as food, are shared, highly depends on how individuals interact with one another (Gordon, 2010). Thus, it is important to determine whether the type of resources and their availability impact the structure of social interactions to shape resource sharing in cooperative groups. Here, we investigate how the structure of interaction networks that facilitate food distribution respond to the type and availability of resources, in the absence of central control.

Resource availability impacts how individuals interact. For example, when carbohydrates are limited, animals increase their foraging for sugars (Hendriksma et al., 2019; Kay, 2004) and similarly, they increase their intake of proteins and other macronutrients when those are limited (Kohl et al., 2015; Mayntz et al., 2005). Limitations on resources may alter how they are shared among group members and therefore such limitations can impact the way in which individuals interact (Hadjichrysanthou and Broom, 2012). Abundant food resources may reduce the need for food-sharing if each individual is able to supply itself adequately, potentially resulting in fewer interactions overall. Similarly, if food supply is limited, we might still expect few interactions if sharing a scarce resource means that each group member will not receive enough to survive.

Furthermore, the types of resources that are distributed can influence the way in which individuals interact. Some resources can be stored and their dissemination does not rely on rapid sharing, while perishable resources that cannot be stored may need to be distributed rapidly. Animals use different types of nutrients to serve different physiological needs, which can change over time (Simpson and Raubenheimer, 2012). For example, when offspring are being produced, ants require and forage for more proteins than when they do not have offspring to feed (Cassill and Tschinkel, 1995). Thus, it is possible that the type of resource that is being shared in a group might determine how it is shared, altering network structure. For example, the presence of perishable goods may result in more interactions to facilitate rapid resource dissemination, compared to when storable resources are being distributed.

Finally, group size is an important factor that shapes patterns of social interaction. Group and colony size have been shown to buffer adverse effects of environmental stressors (reviewed by Linksvayer and Janssen, 2009 and Straub et al., 2015) including food limitations (Kaspari and Vargo, 1995). The more individuals in a group, the greater the potential to interact (Quque et al., 2021). However, increased interactions can be costly, for example, individuals may be at greater risk of exposure to pathogens and injury due to aggression. One way to mitigate such costs may be to subdivide large groups into clusters in which interactions occur more intensely than across clusters (Silk and Fefferman, 2021). Thus, as a group becomes larger, global changes to group structure, or to the way in which a group is organized, may alter how individuals interact (Miller et al., 2022). Groups of different sizes may have different interaction patterns that maintain certain network features (O'Donnell and Bulova, 2007; Pacala et al., 1996). For example, if the number of interactions increases with group size, network density (the number of observed connections over the number of possible connections) may remain constant across group sizes if network density is important for the robustness of resource flow. Alternatively, if the number of interactions does not scale with group size, network density may decrease with group size, potentially slowing down the transfer of goods in larger groups. Here, we ask how group size impacts the structure of a network that is used to transfer resources.

Ants are an ideal model system for examining the dynamics of social interactions because of the important function that interactions have for the fitness of the colony. The way in which ants interact with one another allows them to regulate their collective foraging (Gordon, 2010; Greene and Gordon, 2006; Greene et al., 2013; Pinter-Wollman et al., 2013), for example in response to colony nutritional needs (Csata et al., 2020; Dussutour and Simpson, 2008, 2009). Certain individuals (i.e. foragers) leave the nest to collect food and bring it back to the nest (Gordon, 1989, 1996). Once at the nest, food is distributed and stored, and foragers decide whether or not to continue foraging based on certain types of interactions with nestmates (Miller and Pinter-Wollman, 2023), the forager's own food load (Greenwald et al., 2018; Howard and Tschinkel, 1980; Wallis, 1964), how deep a forager moves into the nest (Baltiansky et al., 2023), and the presence of larvae in the nest (Ulrich et al., 2016).

The sharing of liquid food, which many ant species forage for and consume, is carried out through trophallaxis, which is a mouth-to-mouth interaction in which liquid food is transmitted from one individual to another. Resource availability impacts the speed of food dissemination through the colony: the longer a colony is starved, the quicker newly discovered food is distributed throughout the colony (Howard and Tschinkel, 1981). This change in food distribution speed might be a result of changes to the interaction network that facilitates food sharing (Sendova-Franks et al., 2010). Furthermore, ant colonies require different types of nutrients, with workers primarily consuming sugars, and proteins being consumed by queens and larvae (Markin, 1970). Proteins have a negative impact on worker longevity (Dussutour and Simpson, 2012) and ant foraging decisions are impacted by the type of food they require (Barbee and Pinter-Wollman, 2022; Portha et al., 2002). Because sugars are consumed by workers, they can be viewed as a perishable resource that is not stored, while proteins can be stored in the brood. Sugars are distributed faster among workers than proteins (Howard and Tschinkel, 1981), potentially because ants have more interactions when fed with sugars than when fed with protein. Finally, most food transmission inside the nest occurs among workers, rather than to the queen or larvae, (Wilson and Eisner, 1957) and the number of trophallaxis interactions can increase with group size (Quque et al., 2021). Therefore, group size can impact the way in which food is distributed by altering interaction patterns. Given the ways in which ants respond to food availability, the array of nutritional needs within the colony, and the importance of group size for interactions, ants are an excellent system for examining how the nature and utility of different resources may shape the ways in which resources are distributed.

By studying the interactions among carpenter ants (*Camponotus fragilis*) under different conditions, we ask if social interactions are impacted by group size, food availability, and food type. We quantified interactions using both global- and individual-level network measures and predicted that larger groups would have more interactions to facilitate the transfer of resources. Furthermore, when food supply is unlimited, we expected fewer interactions than when supply is limited if distribution is less important for accessing resources. However, we also expected that when food supply is limited, there would be a decrease in the number of interactions if it is suboptimal to share the scarce resource throughout the entire group (i.e. if each group member will not receive sufficient resources). However, clustering may increase when supply is low relative to when it is unlimited to ensure that at least some group members receive resources. Finally, we expected that when preferable food sources were provided (carbohydrates that are consumed by the workers that collect the food), there would be more interactions and less subdivision of the network (i.e. fewer clusters), compared to when a less preferred resource is provided (protein, which is only consumed by brood), to expedite the flow of the preferred resource.

## RESULTS

Group size, food type, and food availability differed in their impact on the four measures of social behavior. Of the group-level measures, only the number of clusters was impacted by our experimental treatments, but both individual-level centrality measures (degree and betweenness) were impacted by the different experimental manipulations.

Network density was not impacted by group size, food type, food availability, or frame rate. None of the effects in the model were statistically significant (Table 2). The random effect ‘group ID’ explained 13.6% of the variance in the model.

**Table 1.**
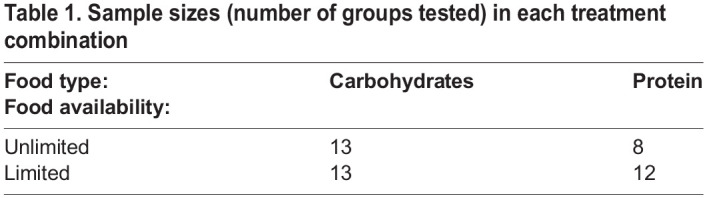
Sample sizes (number of groups tested) in each treatment combination

**Table 2.**
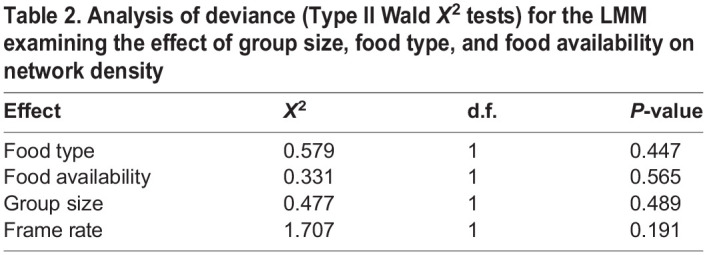
Analysis of deviance (Type II Wald *X*^2^ tests) for the LMM examining the effect of group size, food type, and food availability on network density

The number of clusters in a network was significantly impacted by group size (Table 3). Larger groups had significantly more clusters (Fig. 2, Table 3). The random effect ‘group ID’ explained 28.8% of the variance in the model.

**Fig. 1.**
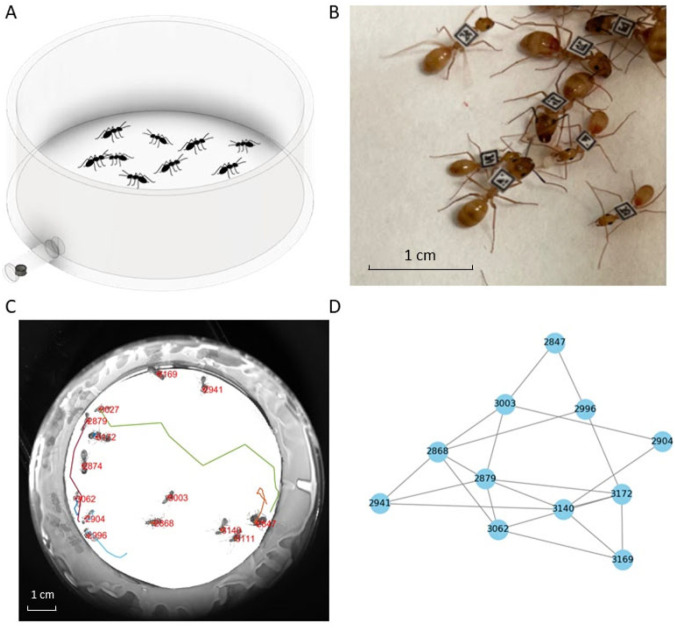
**Experimental setup.** (A) Illustration of the Petri dish in which ants were tracked during the experiments, with the tube that led to the food source at the bottom left (ants not to scale). (B) Image of ant workers (*C. fragilis*) with individually attached BEEtags. (C) An image from one of the experiments overlaid with ants’ trajectories and their individual IDs - as identified from their BEEtags. (D) A social network inferred from imaging data; nodes are individual ants that are connected with edges if their heads came close enough to one another to allow for trophallaxis.

**Fig. 2.**
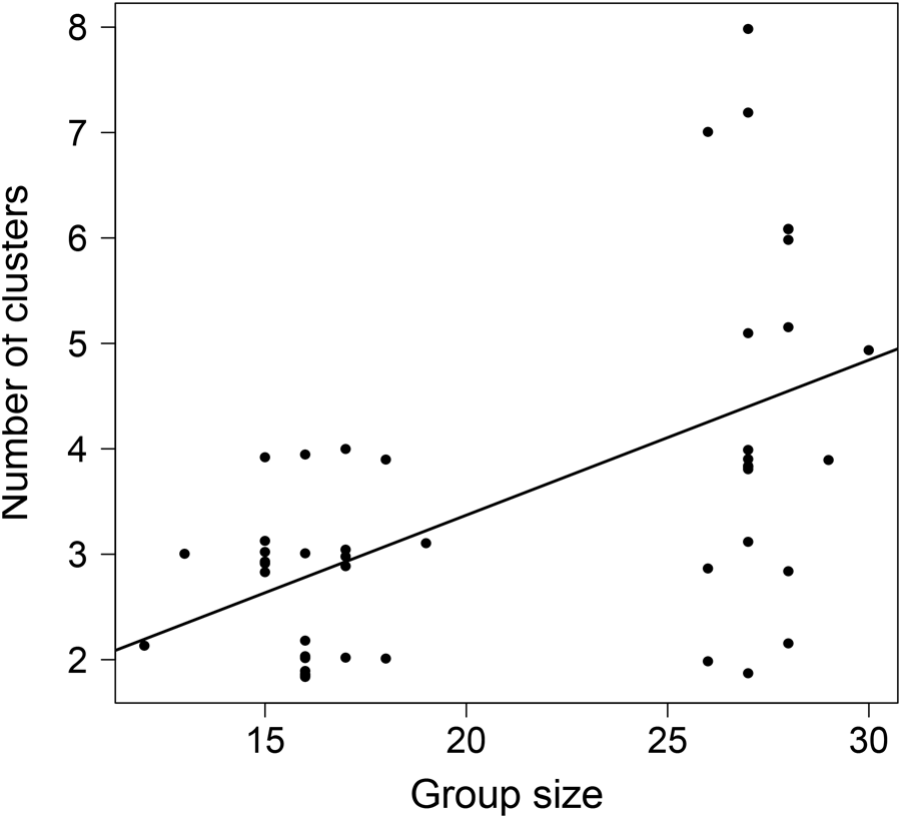
**Number of clusters in the interaction network relates to group size.** *n*=46 groups, see statistics in Table 3. Points are slightly jittered along the y-axis to improve visibility and the line shows the model fit.

**Table 3.**
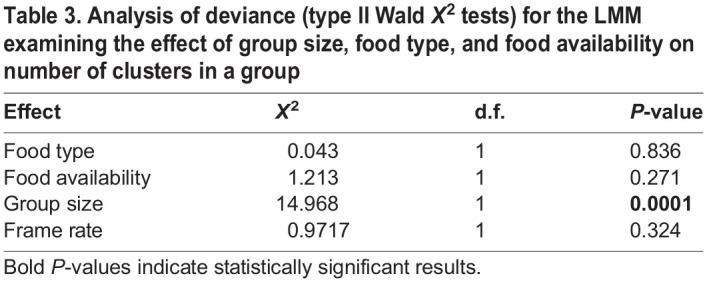
Analysis of deviance (type II Wald *X*^2^ tests) for the LMM examining the effect of group size, food type, and food availability on number of clusters in a group

**Fig. 3.**
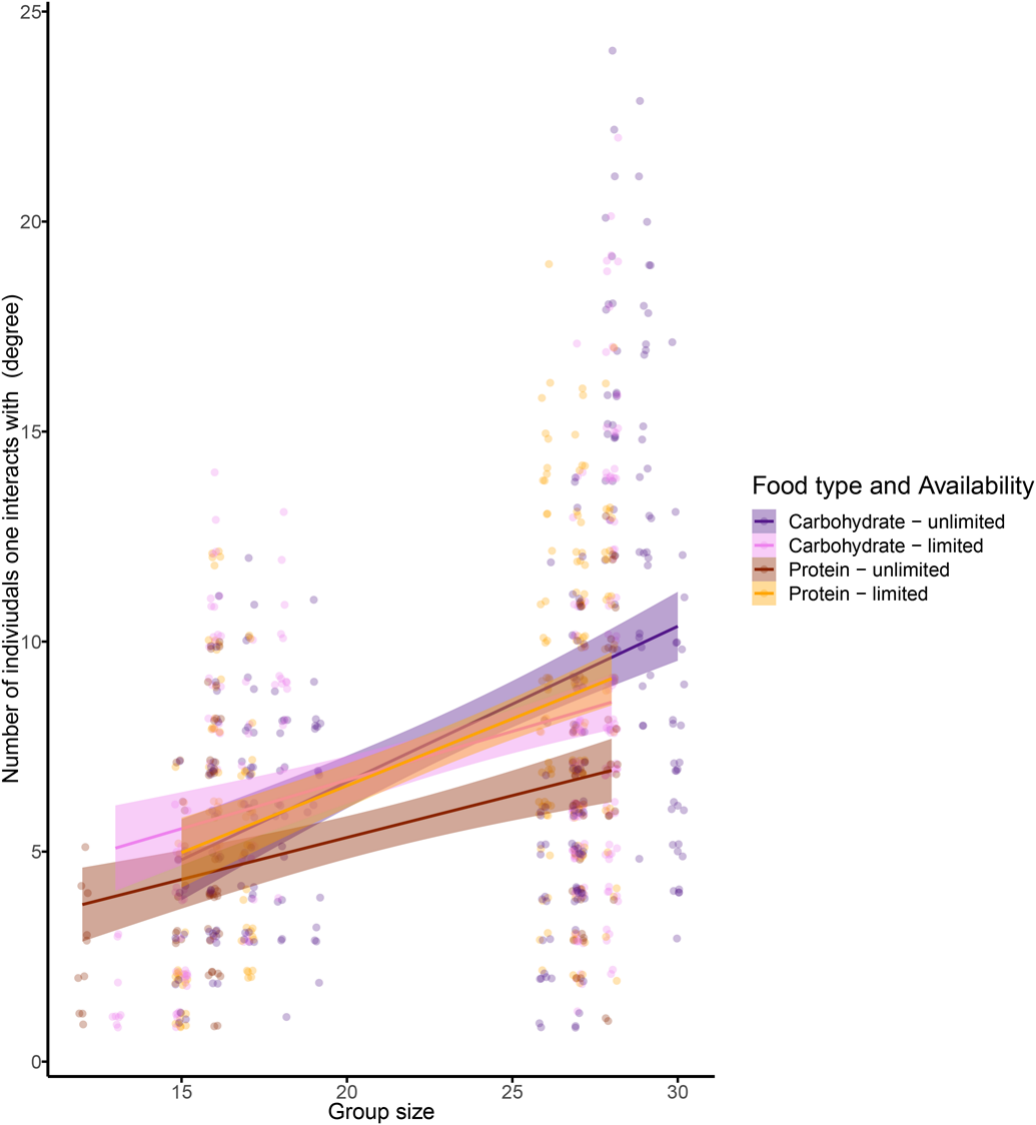
**Number of unique individuals that an ant interacted with (degree) in relation to group size, food type, and food availability.** Each point represents an individual ant (*n*=859 data points from 232 unique individuals and 46 trials, see Table 1 for trial distribution across treatments). Points in purple are from experiments in which the food type was carbohydrate-rich and orange points are from experiments in which the food type was protein-rich. Darker and lighter colors respectively denote experiments in which food supply was unlimited and limited. Lines show the model fit with shaded areas as 95% confidence intervals. Points are slightly jittered along the x and y axes to improve visibility.

The number of unique individuals that each ant interacted with (i.e. degree) was significantly impacted by group size, food availability, and the interaction between food type and food availability (Fig. 3, Table 4). Ants in groups that were fed with carbohydrate-rich food interacted with more unique individuals when food supply was limited than when it was unlimited (post hoc Tukey test comparing food availability by food type, for unlimited versus limited food supply when fed with carbohydrates: estimate=−2.072, standard error (s.e.)=0.572, degrees of freedom (d.f.)=409.8, t=−3.623, *P*-value=0.0019). The random effects ‘group ID’ and ‘individual ID’, and ‘frame rate’ explained 56.9% of the variance in the model.

**Table 4.**
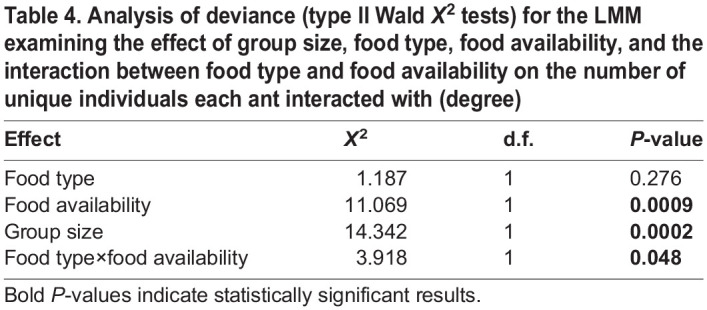
Analysis of deviance (type II Wald *X*^2^ tests) for the LMM examining the effect of group size, food type, food availability, and the interaction between food type and food availability on the number of unique individuals each ant interacted with (degree)

**Fig. 4.**
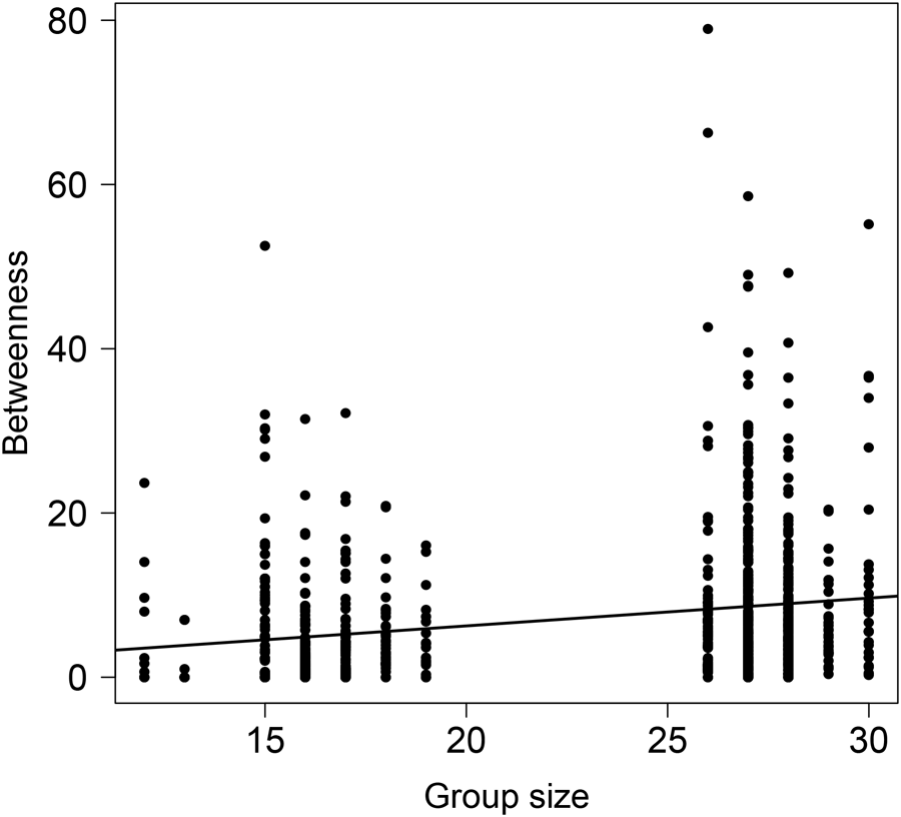
**Betweenness is positively related with group size.** Each point represents an individual ant (*n*=859 data points from 232 unique individuals and 46 trials). Line is the fit of the statistical model.

The number of shortest paths between pairs of ants that pass through a focal ant (i.e. betweenness) was significantly impacted by group size (Fig. 4, Table 5). Individuals in larger groups had significantly higher betweenness (Fig. 4, Table 5).

**Table 5.**
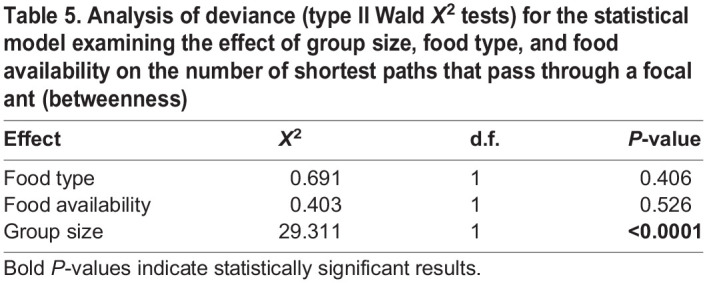
Analysis of deviance (type II Wald *X*^2^ tests) for the statistical model examining the effect of group size, food type, and food availability on the number of shortest paths that pass through a focal ant (betweenness)

**Fig. 5.**
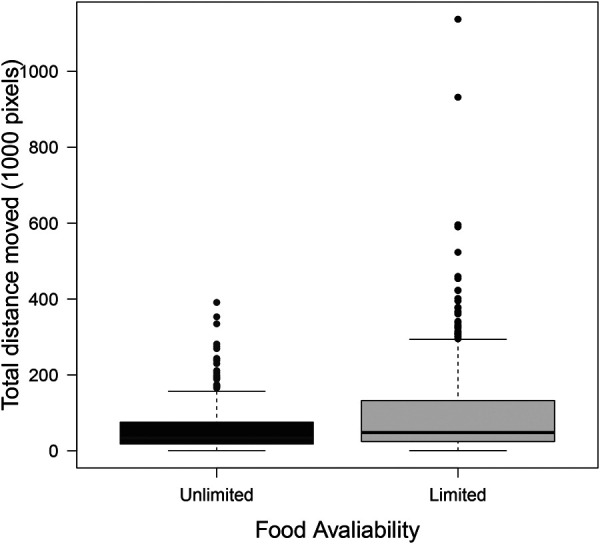
**The total distance that ants moved (displayed in 1000 pixels) throughout the experiment was impacted by food availability.** Ants moved larger distances when food was limited (grey) than when it was unlimited (black). *N*=859 data points from 232 unique individuals and 46 trials see Table 1 for trial distribution across treatments. Horizontal line is the median, boxes extend to 25 and 75 percentiles, whiskers extend to 1.5 times the interquartile range, and points are outliers.

Ant activity was only partially affected by group size, food type, and food availability. Group size and food type did not impact the distance that ants moved, but food availability did (Table 6). When food was limited, ants walked greater distances than when food was unlimited (Fig. 5).

**Table 6.**
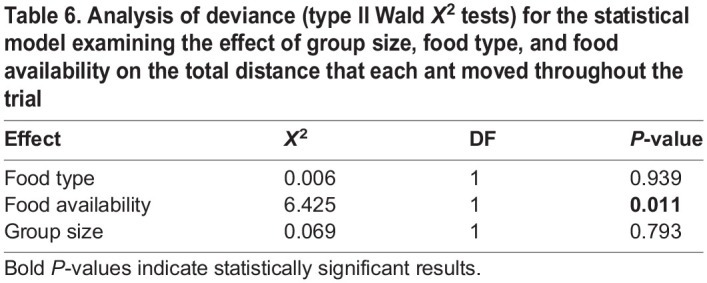
Analysis of deviance (type II Wald *X*^2^ tests) for the statistical model examining the effect of group size, food type, and food availability on the total distance that each ant moved throughout the trial

## DISCUSSION

We found that ant social interactions were impacted by group size and food availability but not by food type. As one might expect, the number of unique individuals contacted by each ant and the number of social clusters in a colony both increased with group size. Interestingly limiting food supply resulted in more interactions with unique individuals and longer distances traveled by ants. These observed differences in network structure have the potential to impact the rate of food transfer among ants because the head-to-head interactions that we observed reflect the potential to engage in food-sharing behaviors.

Individuals in larger groups interacted with more unique individuals (high degree, Fig. 3) and had more shortest paths pass through them (high betweenness, Fig. 4). We further detected more clusters in larger groups (Fig. 2). However, network density, i.e. the observed number of links relative to the possible number of links, did not increase with group size. Because network density is scaled to group size, we can infer that the positive relationship between other network measures and group size emerges simply from having more ants in the group and not from qualitative differences in the way that ants interact when they are in larger groups. The Petri dishes used to house ants in all experiments were the same size, so ants in larger groups were more crowded. Despite this greater crowding of ants in larger groups, network density did not correspond to group size, suggesting that ants maintained a relatively constant interaction rate across group densities (i.e. number of ants per unit area) in the range that we tested. A study of the trophallaxis interactions of the black garden ant (*Lasius niger*) similarly found that the distribution of food throughout the colony is independent of group size (Quque et al., 2021). One way to decouple group size and network density is by reducing the number of ants that participate in interactions. However, we found that most (but not all) ants in a group participated in the interaction networks that we observed (Fig. S1). Only when ants were fed with an unlimited supply of protein-rich food did the proportion of ants that participated in the interaction network slightly decline relative to other treatments (Fig. S2). In addition, maintaining interaction network density across group sizes can be achieved by increasing the number of interactions that each individual experiences with group size. Indeed, we found that as group size increases, ants interact with more unique individuals (Fig. 3), similar to the positive relationship between number of interactions and group size in black garden ants (Quque et al., 2021). Thus, by adjusting the local behavior of each ant (degree), the global structure of the network (network density) is maintained across group sizes and across population densities.

More clustering in larger groups means that there is greater subdivision within an interaction network, increasing the potential for certain individuals to act as ‘brokers’ among clusters, i.e. having higher betweenness. We found that both the number of clusters (Fig. 2) and betweenness (Fig. 4) positively correlated with group size. Work on silky ants (*Formica fusca*) showed that certain individuals in the colony can accumulate food, essentially acting as food storage units that can enhance food distribution across the network (Buffin et al., 2009). While having many clusters may slow down food transfer throughout the entire group, it can also expedite food sharing within clusters, because there are fewer individuals within each cluster (Sah et al., 2017). These dynamics may result in homogeneous food distribution within, but not across, clusters. Such a distribution of interactions might be advantageous to the entire group when supplies are limited as a bet hedging strategy, helping to ensure that at least part of the group (e.g. all individuals in one or more clusters) may have access to a sufficient amount of resources. Without such social sub-division, a limited resource might be distributed evenly across the entire group, risking a situation in which everyone receives too little food to survive. Indeed, within human populations that rely on subsistence harvesting, it has been argued that a modular organization of the population into local resource-sharing clusters can help to mitigate the impacts of systemic resource shortfalls by limiting local collapses (Jones and Ready, 2022). While trophallaxis interactions facilitate a homogenous distribution of cuticular hydrocarbons, which are critical for nestmate recognition (Dahbi et al., 1999), a homogeneous distribution of food to all nestmates might not always be necessary, or advantageous. Our finding that betweenness and number of clusters increased with group size further suggests that subdividing an interaction network may be important for preventing the dilution of a resource across all group members, especially when the group is large. Food availability only impacted the number of unique individuals an ant interacted with (degree) when fed with carbohydrate-rich food and the distance an ant traveled. When food was limited, ants traveled larger distances (Fig. 5) and when they were fed with carbohydrate-rich food, they interacted with more unique individuals (post hoc tests of Fig. 2). It is possible that ants walked more when food was limited in search of food sources. In their search for food, the ants interacted with more unique individuals, potentially engaging in more food-sharing interactions. Indeed, walking patterns have been shown to affect social interactions in ants both using models (Pinter-Wollman, 2015b) and empirical work (Pinter-Wollman et al., 2011).

Food type did not impact any of the measures we examined. The only effect of food type we found was that fewer individuals in the group interacted (were part of the interaction network) when ants were fed with an unlimited supply of protein-rich diet, but there was no effect of food viability on the number of ants participating in the interaction network when fed with carbohydrate-rich diet (Fig. S2). Ant workers consume and use carbohydrates for energy, whereas protein is transferred to the brood and queens (Markin, 1970). In our study, brood was not present in the experimental groups and the protein-rich food still contained approximately half the concentration of sugar as the carbohydrate-rich food (see Supplementary Materials), to ensure that ants eat it. While brood are the main consumers of protein (Howard and Tschinkel, 1981), when providing ants with protein in liquid form mixed with sugars, similar to the protein-rich food used in our study, such food can remain in workers for 24 h (Sorensen and Vinson, 1981). However, the lack of brood in our experimental groups might have resulted in our inability to detect an effect of food type on interaction patterns. Previous work similarly found that the presence of brood does not impact the way in which ants interact (Quque et al., 2021). Thus it is possible that the effect of brood on which food ants consumed is regulated on a longer timescale than the one we studied here. Future work might examine the long-term effects of food type and brood presence on food dissemination throughout an ant colony because carbohydrates cannot be stored and are akin to perishable goods, whereas protein can be stored in larvae and is thus a potential proxy for non-perishable goods. Such differences between perishable and storable goods may reveal differences in interaction structures to facilitate rapid distribution of perishable goods, but other forms of network organization that facilitate acquiring and storing storable goods.

Further work is needed to uncover the mechanisms that underlie the impacts we found of group size and food availability on interaction patterns. For example, changes to the level of activity of workers and their walking patterns when fed different types of food might explain changes in interaction rates (Pinter-Wollman, 2015b). Indeed, we found that the distance ants moved throughout the experiment increased when food was limited, potentially explaining the higher number of unique individuals that ants interacted with (degree) when fed with limited compared to unlimited amounts of carbohydrate-rich food. Perhaps when food was limited, ants were moving around more in search for food, or for full ants to receive food from. Furthermore, the spatial distribution of ants within a nest might determine who interacts with whom and how frequently (Pinter-Wollman, 2015a; Pinter-Wollman et al., 2013, 2011) and can influence foraging decision and the flow of food among nestmates (Baltiansky et al., 2023; Buffin et al., 2009). In our experiment, all trials were conducted in circular Petri dishes with no internal structure. Future work could test the effect of spatial structure on response to food availability and type by testing ants that are housed in different structures. It might also be interesting to determine whether interactions are initiated by fed or hungry ants because recent work suggests that they play different roles in food distribution within ant colonies (Miller and Pinter-Wollman, 2023). In addition, our work did not distinguish between different types of interactions, for example, when ants interact head-to-head, they might be antennating, connecting mandibles without food exchange, or actively exchanging food. Furthermore, ants may have other types of encounters, such as head to body, which may lead to exchange of information through cuticular hydrocarbons, but not to food exchange. While we did not distinguish between different types of interaction, recent work shows that trophallaxis interactions are more important than other interaction types in determining ant foraging decisions (Miller and Pinter-Wollman, 2023). It is possible that some of the patterns we observed here are buffered in large colonies that contain brood. For example, if only a small proportion of the colony participates in interactions to distribute food, the number of individuals engaged in food distribution might be constant and social interactions that facilitate food distribution would not be impacted by the effects of group size that we found here. Indeed, ant group and colony size have been shown to buffer adverse effects of environmental stressors (reviewed by Linksvayer and Janssen, 2009 and Straub et al., 2015) including food limitations (Kaspari and Vargo, 1995). Interestingly, for the three measures in which group size was statistically significant (number of clusters, degree, and betweenness), the best fitting model we identified was also a better fit to the data than a model in which groups size was included as a second-degree polynomial (Tables S7-S9). Thus, ants in larger groups do not change their interactions qualitatively, only quantitatively. The presence of brood that can store (and consume) proteins as well as the negative effects that a protein-rich diet might have on the survival of workers (Dussutour and Simpson, 2012), might further impact the way in which workers interact when provided different types of food. Finally, ant species differ in their propensity to transfer liquid food among workers (Wilson and Eisner, 1957) and a comparative study of food transmission dynamics across ant species might reveal a variety of transmission strategies that are adapted to different environments. To conclude, our work provides insights into how social interactions among ants that can facilitate the exchange of goods relate to group size and to the availability and type of goods. We find more interactions that are more compartmentalized as group sizes increase and as supplies become limited. This adjustment of biological social networks to potentially improve their function may inspire the study and design of robust and resilient supply chains.

## MATERIALS AND METHODS

### Ant maintenance, tagging, and preparation for experiments

We obtained worker ants of the species *Camponotus fragilis* from a colony maintained by an ant supplier (John Truong) on 7 September 2021. After transfer to the lab at UCLA, ants were housed in a rectangular plexiglass container (10.16 cm×5.08 cm×2.54 cm) and fed twice a week with liquid protein-rich food and carbohydrate-rich food (see recipes in the Supplemental Materials). We tagged ants with BEEtags (Crall et al., 2015) that were printed on paper and laminated using transparent Scotch^®^ tape. We used Loctite epoxy adhesive to affix the tags to the ants’ thorax (Fig. 1B). Before each experimental trial, tagged ants were selected randomly and placed together as a group in a Petri dish (90×15 mm) for 5-6 days without food. Group sizes ranged from 14-30 individuals. Note that while we could not find information in the literature on the colony size of this species, other *Camponotus* species have relatively small colony sizes, e.g., colonies of *Camponotus socius* have 150-250 workers (Tschinkel, 2005), *Camponotus japonicus* have approximately 150 workers (Goko et al., 2023), and 1-year old *Camponotus herculaneus* colonies contain less than 15 workers (personal observations from lab-raised colonies). Therefore, the group sizes we examined here compare to young *Camponotus* colonies. All experiments were conducted between 26 October 2021 and 24 June 2022.

### Experimental treatments

Each group of ants was assigned randomly to one of four treatments in which we provided them with either an unlimited or a limited supply of food that was either rich in either carbohydrates or protein. During the experiments, we supplied ants with food outside the Petri dish in which they were housed and tracked (see tracking details below). Liquid food (0.3 ml) was placed in a cap of a microcentrifuge tube which was connected to the Petri dish with a plastic tube (inner diameter: 5 mm, length: 2 cm) that allowed access to the food by only one ant at a time (Fig. 1A).

#### Unlimited food supply

When food supply was unlimited, we provided the food, as detailed above, for the duration of the experiment. We provided enough food so that it did not run out and did not require replenishing during the experiment. While all ants had access to food in these trials, only some ants left the nest to gather food.

#### Limited food supply

When food supply was limited, we provided the ants with food as detailed above until 10% of the unique ants in the group visited the food source, fed, and returned to the tracked Petri dish. Once 10% of the ants fed (this took less than 5 min), we closed the tube connecting the tracked Petri dish to the food using a cotton ball. The time it took 10% of the ants to visit the food was so short that no ant visited the food more than once.

#### Carbohydrate-rich food

We made carbohydrate-rich food by mixing 0.22 g sugar and 2 ml deionized water.

#### Protein-rich food

We made protein-rich food by mixing 0.564 g pasteurized egg powder (Modernist Pantry), 0.188 g sugar and 3.75 ml deionized water.

Both recipes are modified from (Baltiansky et al., 2021). Because workers died during the course of the study, especially when fed with protein, as seen in other studies of ant diet (Dussutour and Simpson, 2012), our experimental design was not completely balanced. For sample sizes in each treatment, see Table 1.

### Filming, image analysis, and inferring interactions

Groups were filmed for 60 min using a camera (FLIR Blackfly, Resolution 5472×3648 PPI) and mounted LED lights for illumination (Thorlabs, MCWHL6) and images were captured using the Micromanager software (Edelstein et al., 2014). In some trials, the time it took to store images while filming reduced the frame rate at certain points in the trial (Fig. S1). Furthermore, the average frame rate across trials was not identical, it ranged from 0.31 to 2.92 images/sec, with an average of 1.61 images/sec and a median of 1.72 images/sec. To account for these differences across trials we included ‘frame rate’ in our statistical models and we did not examine measures that rely on the duration of interactions, only on whether or not an interaction occurred. The position of each ant was extracted from the individually attached BEEtags using a slightly modified BEEtag tracking code in Matlab (Crall et al., 2015) (Fig. 1C), code available on Github (https://github.com/MJHasenjager/Identifying-Trophallaxis-Networks). We used the directionality of the BEEtags to determine the position of each ant's head and considered only head-to-head interactions in our analysis. Considering only head-to-head interactions allowed us to restrict our analysis to interactions that might result in trophallaxis (i.e. exchange of liquid food) and exclude other interactions, such as head-to-abdomen, abdomen-to-abdomen, etc. To identify interactions, we used a distance of 102 pixels (1 pixel: 0.04 mm) as the threshold distance to automatically infer interactions. If the heads of two ants were equal to or less than this threshold distance in any frame, they were considered interacting. We established this threshold based on manual measurements of distances between ants in 50 randomly selected frames from each trial. We averaged the manually measured distances in each trial and used the highest average value for all trials, code available on Github (https://github.com/MJHasenjager/Identifying-Trophallaxis-Networks). Due to variation in frame rates across and within trials, we did not determine the strength (duration) of each interaction, but simply noted whether individuals interacted or not to form an unweighted and undirected interaction network. Note that not all ants interacted and therefore interaction networks might include fewer individuals than the number of individuals in a group (Fig. S1).

### Social network analysis

We used network analysis to quantify the social behavior of the ants and to determine how their social behavior changed in response to the experimental manipulations. Each ant was a node in the network, and an interaction (edge) connected two ants when their heads were within a specified distance threshold (102 pixels) that allowed for trophallaxis, as detailed above (Fig. 1D). To quantify the social behavior of the ants, we used two network measures that quantify global network structure (network density and number of clusters) and two individual-based centrality measures (degree and betweenness). Network measures were calculated using the R package ‘igraph’ (Csardi and Nepusz, 2006).

#### Network density

Number of all observed interactions between ants divided by all possible interactions. This measure provides information about the overall connectivity of the network while scaling for network (group) size.

#### Number of clusters

Number of clusters that each network can be delineated into. We applied the ‘*walktrap*’ clustering algorithm [using the cluster_walktrap() function in the R package ‘igraph’ (Csardi and Nepusz, 2006)] to all networks and recorded the number of clusters that were identified by this algorithm. We selected the ‘*walktrap*’ algorithm, among many available network clustering algorithms, because on visual inspection of the clusters that it identified, it provided the most biologically plausible clusters, in which different clusters were assigned to groups that were least connected.

#### Degree

Number of unique individuals that an ant interacted with. Provides information on how many interaction partners each individual had. This measure is not scaled for group size and so there are often more opportunities to have greater degree values in larger groups.

#### Betweenness

Number of shortest paths that connect pairs of individuals and that pass through the focal ant. This measure provides information on how well each ant acts as a bridge for other ants’ interactions. Individuals with high betweenness connect many ants that might not be well-connected themselves.

### Activity level

To examine whether ant activity changed in response to the experimental manipulations, we measured the ‘total distance moved’ by each ant. We first measured the distance (in pixels) that an ant moved between two consecutive images and then summed all these distances throughout a trial to obtain the ‘total distance moved’ for each ant. This measure was used in other studies to quantify ant activity and it relates to how exploratory an individual is (Page et al., 2018).

### Statistical analysis

To determine the impact of group size, food availability, and food type of social interactions, we ran linear mixed models (LMM) or generalized linear mixed models (GLMM). In each model, one of the network measures (density, number of clusters, degree, or betweenness) or total distance moved was the dependent variable. The explanatory variables included group size (a continuous numeric value), food availability (limited or unlimited), and food type (carbohydrates or protein). For group-level network measures, each data point was for an entire group and we included ‘group ID’ as a random effect because while most groups were used only once, some groups were used multiple times (median three times, see data provided with the analysis code for details on repeated measures). Furthermore, because frame rate slightly differed across trials (Fig. S2) we include the average frame rate of each trial as an effect in the model. For individual-based network measures and for total distance moved each data point was for an individual ant, and we included ‘group ID’, ‘individual ID’, and ‘frame rate’ as random effects in the models to account for variation among groups and among individuals that were tested multiple times in different treatments and to account for variation in frame rate across trials.

We used a model selection approach to determine whether to include interactions among effects in our final statistical model. We ran each model with either no interactions among group size, food type, and food availability; with the three-way interaction term among the three variables; and three additional models with just one interaction each between a different pair of variables each time, totaling five statistical models. We then compared the models using AIC and selected the best fit model, i.e., the one with the lowest AIC score. The best fit models for the two global-level measures, density and number of clusters, as well as for the individual-level network measure ‘betweenness’, and for ‘total distance moved’, included no interaction terms among the explanatory effects. The best fit models for the individual-level network measure ‘degree’ included only one interaction term between food type and food availability (see Tables S2-S6 for AIC values of all the models we tested). All the best fitting models met the required statistical assumptions, examined using the check_model() function in the ‘performance’ package (Lüdecke et al., 2021).

For density, number of clusters, degree, and total distance moved we ran an LMM, implemented using the lmer() function in the ‘lme4’ package. For betweenness, we used a GLMM with a gamma log link function, implemented using the glmer() function in the ‘lme4’ package (Bates et al., 2015). We report the analysis of deviance of the models, obtained using the Anova() function in the ‘car’ R package (Fox and Weisberg, 2019). We report the percent variance explained by the random effects as the conditional R^2^ minus the marginal R^2^. For the only model that included an interaction term (degree), we conducted post hoc Tukey tests using the emmeans() function in the ‘emmeans’ R package (Lenth, 2022).

Image analysis was conducted in Matlab (Mathworks Inc., Natick, MA, USA) and network and statistical analyses were conducted in R (R Core, 2022).

### Data accessibility

All data and code can be found on Github (https://github.com/MJHasenjager/Identifying-Trophallaxis-Networks).

### Ethical Note

This work was conducted in accordance with the local animal welfare laws, guidelines and policy for the use of animals in research. Ants are invertebrates and do not require special institutional permissions for experimentation. We handled ants with extreme care. We used soft tweezers when handling the ants to minimize harm. Experiments involved video recording of ants' behavior, with no invasive methods. After the experiments we kept the ants in the lab and provided them with food *ad lib* until they died naturally.

## Supplementary Material

10.1242/biolopen.060422_sup1 Supplementary information
