# Supplementary material for: Social interactions among ants are impacted by food availability and group size
Source: Biol Open. 2024 Oct 16;13(10):bio060422. doi: 10.1242/bio.060422 (PMC11554260; doi:10.1242/bio.060422)
Supplement: Supplementary information [file biolopen-13-060422-s1.pdf]

Supplementary Materials and Methods

1. Recipe for protein-rich liquid food (0.146M sugar)
3ml Hummingbird nectar concentrate (Perky-Pet)
15 ml DI water
0.1 gram Pasteurized whole Egg powder (Modernist Pantry)

2. Recipe for Carbohydrate-rich liquid food (0.321M sugar)
2ml Hummingbird nectar concentrate
10ml DI water

We fed each group with both sources of food: 0.5ml protein-rich liquid food in one tube and 1ml carbohydrate-rich liquid food in another tube. We replaced the protein-rich liquid food twice per week, and the carbohydrate-rich food once per week.

Biology Open • Supplementary information

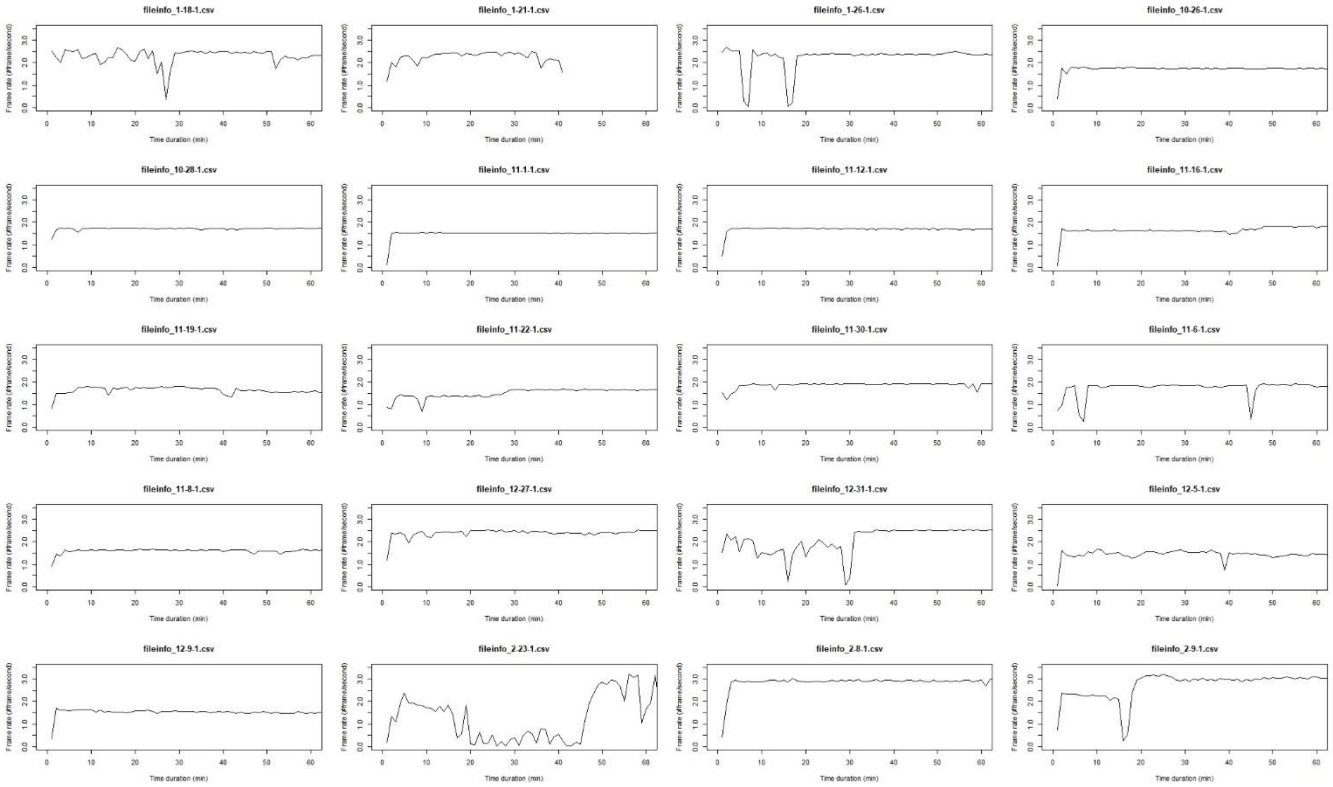
fileinfo_1-18-1.csv
fileinfo_1-21-1.csv
fileinfo_1-26-1.csv
fileinfo_10-26-1.csv
fileinfo_10-28-1.csv
fileinfo_11-1-1.csv
fileinfo_11-12-1.csv
fileinfo_11-16-1.csv
fileinfo_11-19-1.csv
fileinfo_11-22-1.csv
fileinfo_11-30-1.csv
fileinfo_11-6-1.csv
fileinfo_11-8-1.csv
fileinfo_12-27-1.csv
fileinfo_12-31-1.csv
fileinfo_12-5-1.csv
fileinfo_12-9-1.csv
fileinfo_2-23-1.csv
fileinfo_2-8-1.csv
fileinfo_2-9-1.csv
Frame rate (#frame/second)
Time duration (min)

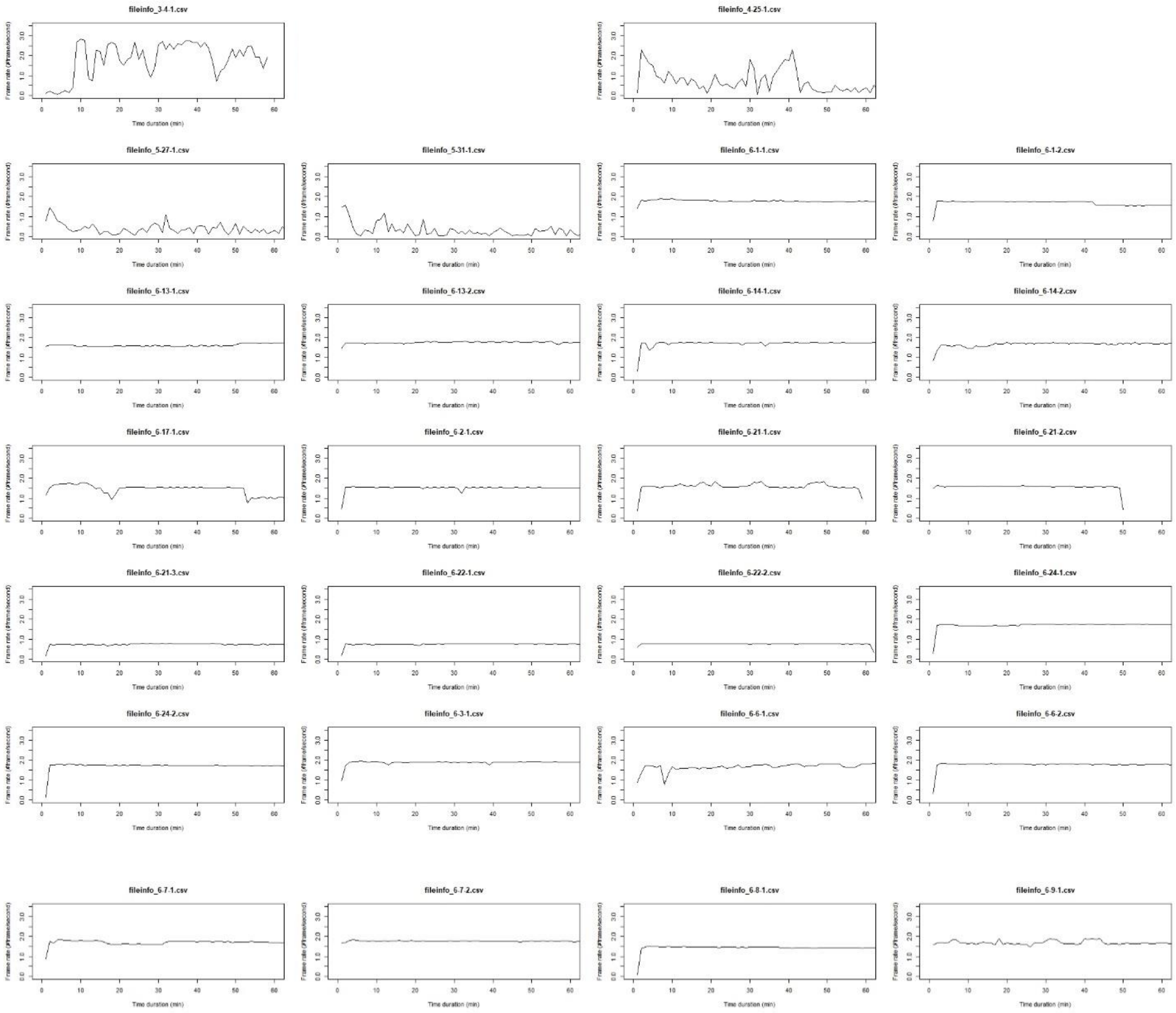

Fig. S1. Frame rate (frames/sec) over time for all trials – each plot is for a different trial.

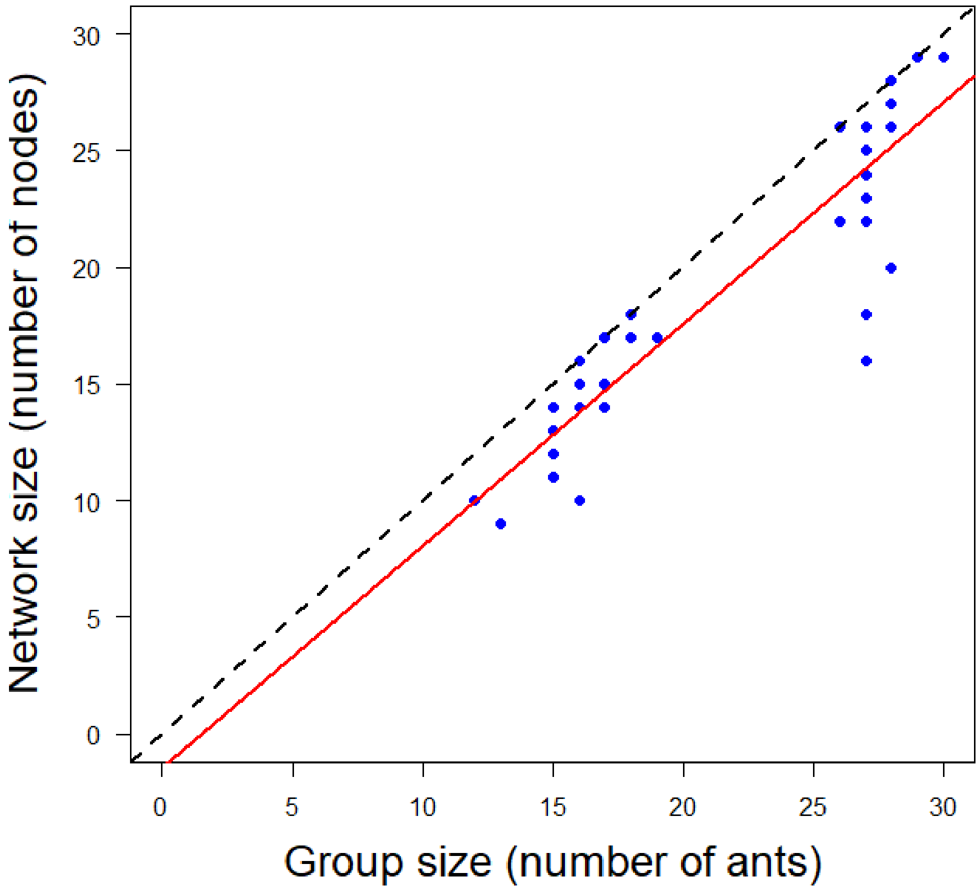

Fig. S2. Relationship between the number of ants in a group (x axis) and the number of ants participating in the interaction network (y axis). The relationship between the two is plotted as a solid red line (Pearson's correlation: r=0.917, p<0.0001). A one-to-one relationship is plotted as a dashed black line for comparison.

Biology Open • Supplementary information

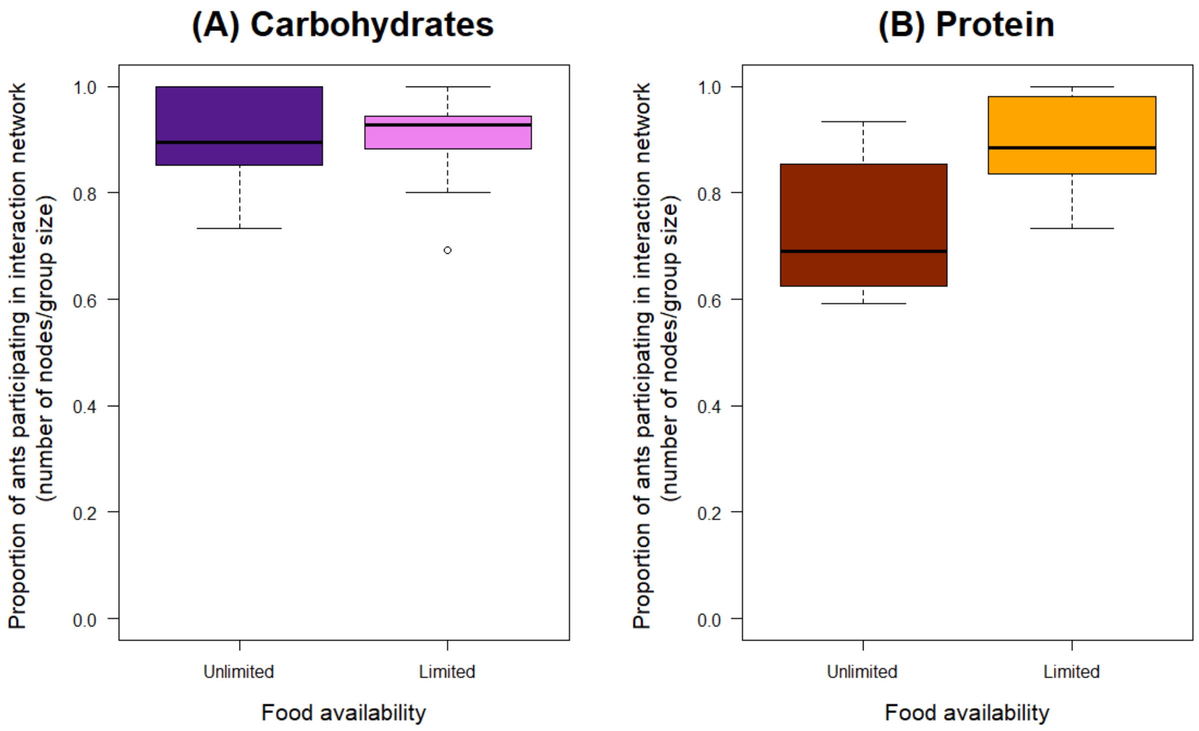

Fig. S3. The proportion of ants participating in social interactions (number of nodes in the network divided by number of ants in the group) was close to 1 when ants were fed with carbohydrate-rich food and a limited supply of protein-rich food. However, when they were fed with an unlimited supply of protein-rich food, not all ants participated in the interaction network.

Table S1. Analysis of Deviance Table for the proportion of ants participation in the interaction network as a function of food availability and food type:

**Effect**	X^2	**DF**	**p-value**
Food type	5.771	1	**0.016**
Food availability	3.553	1	0.059
Food type x Food availability	9.239	1	**0.002**

Biology Open • Supplementary information

Model comparisons: Best fit model (lowest AIC value) is in bold.

Table S2. Density.

Model	AIC
FoodType * Treatment * GroupSize + Frame_rate + (1|Group.ID)	-40.0
**FoodType + Treatment + GroupSize + Frame_rate + (1|Group.ID)**	**-45.8**
FoodType * Treatment + GroupSize + Frame_rate + (1|Group.ID)	-44.3
FoodType + Treatment * GroupSize + Frame_rate + (1|Group.ID)	-44.1
FoodType * GroupSize + Treatment + Frame_rate + (1|Group.ID)	-44.1

Table S3. Number of clusters.

Model	AIC
FoodType * Treatment * GroupSize + Frame_rate + (1|Group.ID)	166.9
**FoodType + Treatment + GroupSize + Frame_rate + (1|Group.ID)**	**161.4**
FoodType * Treatment + GroupSize + Frame_rate + (1|Group.ID)	162.3
FoodType + Treatment * GroupSize + Frame_rate + (1|Group.ID)	163.2
FoodType * GroupSize + Treatment + Frame_rate + (1|Group.ID)	163.2

Table S4. Degree

Model	AIC
FoodType * Treatment * GroupSize + (1|Group.ID) + (1|IndividualID) + (1|Frame_rate)	4361.8
FoodType + Treatment + GroupSize + (1|Group.ID) + (1|IndividualID) + (1|Frame_rate)	4357.3
**FoodType * Treatment + GroupSize + (1|Group.ID) + (1|IndividualID) + (1|Frame_rate)**	**4355.4**
FoodType + Treatment * GroupSize + (1|Group.ID) + (1|IndividualID) + (1|Frame_rate)	4359.2
FoodType * GroupSize + Treatment + (1|Group.ID) + (1|IndividualID) + (1|Frame_rate)	4358.5

Table S5. Betweenness

Model	AIC
FoodType * Treatment * GroupSize + (1|Group.ID) + (1|IndividualID) + (1|Frame_rate)	4532.4
**FoodType + Treatment + GroupSize + (1|Group.ID) + (1|IndividualID) + (1|Frame_rate)**	**4527.4**
FoodType * Treatment + GroupSize + (1|Group.ID) + (1|IndividualID) + (1|Frame_rate)	4528.8
FoodType + Treatment * GroupSize + (1|Group.ID) + (1|IndividualID) + (1|Frame_rate)	4528.8
FoodType * GroupSize + Treatment + (1|Group.ID) + (1|IndividualID) + (1|Frame_rate)	4528.4

Table S6. Total distance traveled

Model	AIC
FoodType * Treatment * GroupSize + (1|Group.ID) + (1|IndividualID) + (1|Frame_rate)	22056.5
**FoodType + Treatment + GroupSize + (1|Group.ID) + (1|IndividualID) + (1|Frame_rate)**	**22050.6**
FoodType * Treatment + GroupSize + (1|Group.ID) + (1|IndividualID) + (1|Frame_rate)	22051.8
FoodType + Treatment * GroupSize + (1|Group.ID) + (1|IndividualID) + (1|Frame_rate)	22051.7
FoodType * GroupSize + Treatment + (1|Group.ID) + (1|IndividualID) + (1|Frame_rate)	22052.4

Table S7. Comparing models for number of clusters with a linear or polynomial fit to group size

Model	AIC
**FoodType + Treatment + GroupSize + Frame_rate + (1|Group.ID)**	**161.4**
FoodType + Treatment + GroupSize + GroupSize^2 + Frame_rate + (1|Group.ID)	162.9

Table S8. Comparing models for degree with a linear or polynomial fit to group size

Model	AIC
**FoodType * Treatment + GroupSize + (1|Group.ID) + (1|IndividualID) + (1|Frame_rate)**	**4355.4**
FoodType * Treatment + GroupSize + GroupSize^2 + (1|Group.ID) + (1|IndividualID) + (1|Frame_rate)	4357

Table S9. Comparing models for betweeness with a linear or polynomial fit to group size

Model	AIC
**FoodType + Treatment + GroupSize + (1|Group.ID) + (1|IndividualID) + (1|Frame_rate)**	**4527.4**
FoodType + Treatment + GroupSize + GroupSize^2 + (1|Group.ID) + (1|IndividualID) + (1|Frame_rate)	4528.7

Biology Open • Supplementary information
